# Supplementary material for: HIVprotI: an integrated web based platform for prediction and design of HIV proteins inhibitors
Source: J Cheminform. 2018 Mar 9;10:12. doi: 10.1186/s13321-018-0266-y (PMC5845081; doi:10.1186/s13321-018-0266-y)
Supplement: Supplementary file 2 — Additional file 2. Source code of HIVProtI web server. [file 13321_2018_266_MOESM2_ESM.zip › HIVprotI_Source-code/hivprotI/weka-3-6-6/doc/weka/gui/arffviewer/ArffPanel.html]

ArffPanel


---


|  |  |  |
| --- | --- | --- |
| **Overview** \| **Package** \| **Class** \| **Tree** \| **Deprecated** \| **Index** \| **Help** \| **Weka's home** | |  |
| PREV CLASS   **NEXT CLASS** | **FRAMES**    **NO FRAMES**     **All Classes** |  |
| SUMMARY: NESTED \| FIELD \| CONSTR \| METHOD | DETAIL: FIELD \| CONSTR \| METHOD |  |


---


## weka.gui.arffviewer Class ArffPanel

```
java.lang.Object
  java.awt.Component
      java.awt.Container
          javax.swing.JComponent
              javax.swing.JPanel
                  weka.gui.arffviewer.ArffPanel
```

**All Implemented Interfaces:**: java.awt.event.ActionListener, java.awt.event.MouseListener, java.awt.image.ImageObserver, java.awt.MenuContainer, java.io.Serializable, java.util.EventListener, javax.accessibility.Accessible, javax.swing.event.ChangeListener, Undoable

---

``` public class ArffPanel extends javax.swing.JPanel implements java.awt.event.ActionListener, javax.swing.event.ChangeListener, java.awt.event.MouseListener, Undoable ```

A Panel representing an ARFF-Table and the associated filename.

**Version:**
:   $Revision: 7369 $

**Author:**
:   FracPete (fracpete at waikato dot ac dot nz)

**See Also:**: Serialized Form

---

**Nested Class Summary**

**Nested classes/interfaces inherited from class javax.swing.JComponent**

`javax.swing.JComponent.AccessibleJComponent`

**Nested classes/interfaces inherited from class java.awt.Component**

`java.awt.Component.BaselineResizeBehavior`


| **Field Summary** | |
| --- | --- |
| `static java.lang.String` | `TAB_INSTANCES`             the name of the tab for instances that were set directly |

**Fields inherited from class javax.swing.JComponent**

`TOOL_TIP_TEXT_KEY, UNDEFINED_CONDITION, WHEN_ANCESTOR_OF_FOCUSED_COMPONENT, WHEN_FOCUSED, WHEN_IN_FOCUSED_WINDOW`

**Fields inherited from class java.awt.Component**

`BOTTOM_ALIGNMENT, CENTER_ALIGNMENT, LEFT_ALIGNMENT, RIGHT_ALIGNMENT, TOP_ALIGNMENT`

**Fields inherited from interface java.awt.image.ImageObserver**

`ABORT, ALLBITS, ERROR, FRAMEBITS, HEIGHT, PROPERTIES, SOMEBITS, WIDTH`


**Constructor Summary**

`ArffPanel()`
  
          initializes the panel with no data

`ArffPanel(Instances data)`
  
          initializes the panel with the given data

`ArffPanel(java.lang.String filename)`
  
          initializes the panel and loads the specified file


| **Method Summary** | |
| --- | --- |
| `void` | `actionPerformed(java.awt.event.ActionEvent e)`             invoked when an action occurs |
| `void` | `addChangeListener(javax.swing.event.ChangeListener l)`             Adds a ChangeListener to the panel |
| `void` | `addUndoPoint()`             adds the current state of the instances to the undolist |
| `void` | `attributeAsClass()`             sets the current attribute as class attribute, i.e. |
| `boolean` | `canUndo()`             returns whether an undo is possible |
| `void` | `clearSearch()`             clears the search, i.e. |
| `void` | `clearUndo()`             removes the undo history |
| `void` | `copyContent()`             copies the content of the selection to the clipboard |
| `void` | `deleteAttribute()`             deletes the currently selected attribute |
| `void` | `deleteAttributes()`             deletes the chosen attributes |
| `void` | `deleteInstance()`             deletes the currently selected instance |
| `void` | `deleteInstances()`             deletes all the currently selected instances |
| `java.util.Vector` | `getAttributes()`             returns a list with the attributes |
| `java.lang.String` | `getFilename()`             returns the filename |
| `Instances` | `getInstances()`             returns the instances of the panel, if none then NULL |
| `ArffTable` | `getTable()`             returns the table component |
| `java.lang.String` | `getTitle()`             returns the title for the Tab, i.e. |
| `boolean` | `isChanged()`             returns whether the content of the panel was changed |
| `boolean` | `isReadOnly()`             returns whether the model is read-only |
| `boolean` | `isUndoEnabled()`             returns whether undo support is enabled |
| `void` | `mouseClicked(java.awt.event.MouseEvent e)`             Invoked when a mouse button has been pressed and released on a component |
| `void` | `mouseEntered(java.awt.event.MouseEvent e)`             Invoked when the mouse enters a component. |
| `void` | `mouseExited(java.awt.event.MouseEvent e)`             Invoked when the mouse exits a component |
| `void` | `mousePressed(java.awt.event.MouseEvent e)`             Invoked when a mouse button has been pressed on a component |
| `void` | `mouseReleased(java.awt.event.MouseEvent e)`             Invoked when a mouse button has been released on a component. |
| `void` | `notifyListener()`             notfies all listener of the change |
| `void` | `removeChangeListener(javax.swing.event.ChangeListener l)`             Removes a ChangeListener from the panel |
| `void` | `renameAttribute()`             renames the current attribute |
| `void` | `search()`             searches for a string in the cells |
| `void` | `setChanged(boolean changed)`             can only reset the changed state to FALSE |
| `void` | `setFilename(java.lang.String filename)`             sets the filename |
| `void` | `setInstances(Instances data)`             displays the given instances, i.e. |
| `void` | `setOptimalColWidth()`             calculates the optimal column width for the current column |
| `void` | `setOptimalColWidths()`             calculates the optimal column widths for all columns |
| `void` | `setReadOnly(boolean value)`             sets whether the model is read-only |
| `void` | `setUndoEnabled(boolean enabled)`             sets whether undo support is enabled |
| `void` | `sortInstances()`             sorts the instances via the currently selected column |
| `void` | `stateChanged(javax.swing.event.ChangeEvent e)`             Invoked when the target of the listener has changed its state. |
| `void` | `undo()`             performs an undo action |

**Methods inherited from class javax.swing.JPanel**

`getAccessibleContext, getUI, getUIClassID, setUI, updateUI`

**Methods inherited from class javax.swing.JComponent**

`addAncestorListener, addNotify, addVetoableChangeListener, computeVisibleRect, contains, createToolTip, disable, enable, firePropertyChange, firePropertyChange, firePropertyChange, getActionForKeyStroke, getActionMap, getAlignmentX, getAlignmentY, getAncestorListeners, getAutoscrolls, getBaseline, getBaselineResizeBehavior, getBorder, getBounds, getClientProperty, getComponentPopupMenu, getConditionForKeyStroke, getDebugGraphicsOptions, getDefaultLocale, getFontMetrics, getGraphics, getHeight, getInheritsPopupMenu, getInputMap, getInputMap, getInputVerifier, getInsets, getInsets, getListeners, getLocation, getMaximumSize, getMinimumSize, getNextFocusableComponent, getPopupLocation, getPreferredSize, getRegisteredKeyStrokes, getRootPane, getSize, getToolTipLocation, getToolTipText, getToolTipText, getTopLevelAncestor, getTransferHandler, getVerifyInputWhenFocusTarget, getVetoableChangeListeners, getVisibleRect, getWidth, getX, getY, grabFocus, isDoubleBuffered, isLightweightComponent, isManagingFocus, isOpaque, isOptimizedDrawingEnabled, isPaintingForPrint, isPaintingTile, isRequestFocusEnabled, isValidateRoot, paint, paintImmediately, paintImmediately, print, printAll, putClientProperty, registerKeyboardAction, registerKeyboardAction, removeAncestorListener, removeNotify, removeVetoableChangeListener, repaint, repaint, requestDefaultFocus, requestFocus, requestFocus, requestFocusInWindow, resetKeyboardActions, reshape, revalidate, scrollRectToVisible, setActionMap, setAlignmentX, setAlignmentY, setAutoscrolls, setBackground, setBorder, setComponentPopupMenu, setDebugGraphicsOptions, setDefaultLocale, setDoubleBuffered, setEnabled, setFocusTraversalKeys, setFont, setForeground, setInheritsPopupMenu, setInputMap, setInputVerifier, setMaximumSize, setMinimumSize, setNextFocusableComponent, setOpaque, setPreferredSize, setRequestFocusEnabled, setToolTipText, setTransferHandler, setVerifyInputWhenFocusTarget, setVisible, unregisterKeyboardAction, update`

**Methods inherited from class java.awt.Container**

`add, add, add, add, add, addContainerListener, addPropertyChangeListener, addPropertyChangeListener, applyComponentOrientation, areFocusTraversalKeysSet, countComponents, deliverEvent, doLayout, findComponentAt, findComponentAt, getComponent, getComponentAt, getComponentAt, getComponentCount, getComponents, getComponentZOrder, getContainerListeners, getFocusTraversalKeys, getFocusTraversalPolicy, getLayout, getMousePosition, insets, invalidate, isAncestorOf, isFocusCycleRoot, isFocusCycleRoot, isFocusTraversalPolicyProvider, isFocusTraversalPolicySet, layout, list, list, locate, minimumSize, paintComponents, preferredSize, printComponents, remove, remove, removeAll, removeContainerListener, setComponentZOrder, setFocusCycleRoot, setFocusTraversalPolicy, setFocusTraversalPolicyProvider, setLayout, transferFocusBackward, transferFocusDownCycle, validate`

**Methods inherited from class java.awt.Component**

`action, add, addComponentListener, addFocusListener, addHierarchyBoundsListener, addHierarchyListener, addInputMethodListener, addKeyListener, addMouseListener, addMouseMotionListener, addMouseWheelListener, bounds, checkImage, checkImage, contains, createImage, createImage, createVolatileImage, createVolatileImage, dispatchEvent, enable, enableInputMethods, firePropertyChange, firePropertyChange, firePropertyChange, firePropertyChange, firePropertyChange, getBackground, getBounds, getColorModel, getComponentListeners, getComponentOrientation, getCursor, getDropTarget, getFocusCycleRootAncestor, getFocusListeners, getFocusTraversalKeysEnabled, getFont, getForeground, getGraphicsConfiguration, getHierarchyBoundsListeners, getHierarchyListeners, getIgnoreRepaint, getInputContext, getInputMethodListeners, getInputMethodRequests, getKeyListeners, getLocale, getLocation, getLocationOnScreen, getMouseListeners, getMouseMotionListeners, getMousePosition, getMouseWheelListeners, getName, getParent, getPeer, getPropertyChangeListeners, getPropertyChangeListeners, getSize, getToolkit, getTreeLock, gotFocus, handleEvent, hasFocus, hide, imageUpdate, inside, isBackgroundSet, isCursorSet, isDisplayable, isEnabled, isFocusable, isFocusOwner, isFocusTraversable, isFontSet, isForegroundSet, isLightweight, isMaximumSizeSet, isMinimumSizeSet, isPreferredSizeSet, isShowing, isValid, isVisible, keyDown, keyUp, list, list, list, location, lostFocus, mouseDown, mouseDrag, mouseEnter, mouseExit, mouseMove, mouseUp, move, nextFocus, paintAll, postEvent, prepareImage, prepareImage, remove, removeComponentListener, removeFocusListener, removeHierarchyBoundsListener, removeHierarchyListener, removeInputMethodListener, removeKeyListener, removeMouseListener, removeMouseMotionListener, removeMouseWheelListener, removePropertyChangeListener, removePropertyChangeListener, repaint, repaint, repaint, resize, resize, setBounds, setBounds, setComponentOrientation, setCursor, setDropTarget, setFocusable, setFocusTraversalKeysEnabled, setIgnoreRepaint, setLocale, setLocation, setLocation, setName, setSize, setSize, show, show, size, toString, transferFocus, transferFocusUpCycle`

**Methods inherited from class java.lang.Object**

`equals, getClass, hashCode, notify, notifyAll, wait, wait, wait`

**Field Detail**

### TAB\_INSTANCES

```
public static final java.lang.String TAB_INSTANCES
```

:   the name of the tab for instances that were set directly


**Constructor Detail**

### ArffPanel

```
public ArffPanel()
```

:   initializes the panel with no data

---


### ArffPanel

```
public ArffPanel(java.lang.String filename)
```

:   initializes the panel and loads the specified file

    **Parameters:**: `filename` - the file to load

---


### ArffPanel

```
public ArffPanel(Instances data)
```

:   initializes the panel with the given data

    **Parameters:**: `data` - the data to use


**Method Detail**

### getTable

```
public ArffTable getTable()
```

:   returns the table component

    :   **Returns:**: the table

---


### getTitle

```
public java.lang.String getTitle()
```

:   returns the title for the Tab, i.e. the filename

    :   **Returns:**: the title for the tab

---


### getFilename

```
public java.lang.String getFilename()
```

:   returns the filename

    :   **Returns:**: the filename

---


### setFilename

```
public void setFilename(java.lang.String filename)
```

:   sets the filename

    :   **Parameters:**: `filename` - the new filename

---


### getInstances

```
public Instances getInstances()
```

:   returns the instances of the panel, if none then NULL

    :   **Returns:**: the instances of the panel

---


### setInstances

```
public void setInstances(Instances data)
```

:   displays the given instances, i.e. creates a tab with the title
    TAB\_INSTANCES. if one already exists it closes it.  
    if a different instances object is used here, don't forget to clear
    the undo-history by calling `clearUndo()`

    :   **Parameters:**: `data` - the instances to display **See Also:**: `TAB_INSTANCES`, `clearUndo()`

---


### getAttributes

```
public java.util.Vector getAttributes()
```

:   returns a list with the attributes

    :   **Returns:**: a list of the attributes

---


### setChanged

```
public void setChanged(boolean changed)
```

:   can only reset the changed state to FALSE

    :   **Parameters:**: `changed` - if false, resets the changed state

---


### isChanged

```
public boolean isChanged()
```

:   returns whether the content of the panel was changed

    :   **Returns:**: true if the content was changed

---


### isReadOnly

```
public boolean isReadOnly()
```

:   returns whether the model is read-only

    :   **Returns:**: true if model is read-only

---


### setReadOnly

```
public void setReadOnly(boolean value)
```

:   sets whether the model is read-only

    :   **Parameters:**: `value` - if true the model is set to read-only

---


### isUndoEnabled

```
public boolean isUndoEnabled()
```

:   returns whether undo support is enabled

    :   **Specified by:**: `isUndoEnabled` in interface `Undoable`
    :   **Returns:**: true if undo is enabled

---


### setUndoEnabled

```
public void setUndoEnabled(boolean enabled)
```

:   sets whether undo support is enabled

    :   **Specified by:**: `setUndoEnabled` in interface `Undoable`
    :   **Parameters:**: `enabled` - whether to enable/disable undo support

---


### clearUndo

```
public void clearUndo()
```

:   removes the undo history

    :   **Specified by:**: `clearUndo` in interface `Undoable`

---


### canUndo

```
public boolean canUndo()
```

:   returns whether an undo is possible

    :   **Specified by:**: `canUndo` in interface `Undoable`
    :   **Returns:**: true if undo is possible

---


### undo

```
public void undo()
```

:   performs an undo action

    :   **Specified by:**: `undo` in interface `Undoable`

---


### addUndoPoint

```
public void addUndoPoint()
```

:   adds the current state of the instances to the undolist

    :   **Specified by:**: `addUndoPoint` in interface `Undoable`

---


### deleteAttribute

```
public void deleteAttribute()
```

:   deletes the currently selected attribute

---


### deleteAttributes

```
public void deleteAttributes()
```

:   deletes the chosen attributes

---


### attributeAsClass

```
public void attributeAsClass()
```

:   sets the current attribute as class attribute, i.e. it moves it to the end
    of the attributes

---


### renameAttribute

```
public void renameAttribute()
```

:   renames the current attribute

---


### deleteInstance

```
public void deleteInstance()
```

:   deletes the currently selected instance

---


### deleteInstances

```
public void deleteInstances()
```

:   deletes all the currently selected instances

---


### sortInstances

```
public void sortInstances()
```

:   sorts the instances via the currently selected column

---


### copyContent

```
public void copyContent()
```

:   copies the content of the selection to the clipboard

---


### search

```
public void search()
```

:   searches for a string in the cells

---


### clearSearch

```
public void clearSearch()
```

:   clears the search, i.e. resets the found cells

---


### setOptimalColWidth

```
public void setOptimalColWidth()
```

:   calculates the optimal column width for the current column

---


### setOptimalColWidths

```
public void setOptimalColWidths()
```

:   calculates the optimal column widths for all columns

---


### actionPerformed

```
public void actionPerformed(java.awt.event.ActionEvent e)
```

:   invoked when an action occurs

    :   **Specified by:**: `actionPerformed` in interface `java.awt.event.ActionListener`
    :   **Parameters:**: `e` - the action event

---


### mouseClicked

```
public void mouseClicked(java.awt.event.MouseEvent e)
```

:   Invoked when a mouse button has been pressed and released on a component

    :   **Specified by:**: `mouseClicked` in interface `java.awt.event.MouseListener`
    :   **Parameters:**: `e` - the mouse event

---


### mouseEntered

```
public void mouseEntered(java.awt.event.MouseEvent e)
```

:   Invoked when the mouse enters a component.

    :   **Specified by:**: `mouseEntered` in interface `java.awt.event.MouseListener`
    :   **Parameters:**: `e` - the mouse event

---


### mouseExited

```
public void mouseExited(java.awt.event.MouseEvent e)
```

:   Invoked when the mouse exits a component

    :   **Specified by:**: `mouseExited` in interface `java.awt.event.MouseListener`
    :   **Parameters:**: `e` - the mouse event

---


### mousePressed

```
public void mousePressed(java.awt.event.MouseEvent e)
```

:   Invoked when a mouse button has been pressed on a component

    :   **Specified by:**: `mousePressed` in interface `java.awt.event.MouseListener`
    :   **Parameters:**: `e` - the mouse event

---


### mouseReleased

```
public void mouseReleased(java.awt.event.MouseEvent e)
```

:   Invoked when a mouse button has been released on a component.

    :   **Specified by:**: `mouseReleased` in interface `java.awt.event.MouseListener`
    :   **Parameters:**: `e` - the mouse event

---


### stateChanged

```
public void stateChanged(javax.swing.event.ChangeEvent e)
```

:   Invoked when the target of the listener has changed its state.

    :   **Specified by:**: `stateChanged` in interface `javax.swing.event.ChangeListener`
    :   **Parameters:**: `e` - the change event

---


### notifyListener

```
public void notifyListener()
```

:   notfies all listener of the change

---


### addChangeListener

```
public void addChangeListener(javax.swing.event.ChangeListener l)
```

:   Adds a ChangeListener to the panel

    :   **Parameters:**: `l` - the listener to add

---


### removeChangeListener

```
public void removeChangeListener(javax.swing.event.ChangeListener l)
```

:   Removes a ChangeListener from the panel

    :   **Parameters:**: `l` - the listener to remove


---


|  |  |  |
| --- | --- | --- |
| **Overview** \| **Package** \| **Class** \| **Tree** \| **Deprecated** \| **Index** \| **Help** \| **Weka's home** | |  |
| PREV CLASS   **NEXT CLASS** | **FRAMES**    **NO FRAMES**     **All Classes** |  |
| SUMMARY: NESTED \| FIELD \| CONSTR \| METHOD | DETAIL: FIELD \| CONSTR \| METHOD |  |


---
